# Supplementary material for: Low-cost, low-input RNA-seq protocols perform nearly as well as high-input protocols
Source: PeerJ. 2015 Mar 26;3:e869. doi: 10.7717/peerj.869 (PMC4380159; doi:10.7717/peerj.869)
Supplement: Text S1 [file peerj-03-869-s001.pdf]

Estimating Complexity of RNAseq Libraries

The presence of a PCR amplification step in most RNA-seq protocols has the potential to introduce a significant number of duplicated reads that arise from the same cDNA fragment (Benjamini and Speed 2012; Xu et al. 2012). Although there are experimental approaches to mitigate this effect (Mamanova et al. 2010), another computational option is to simply remove reads that map to identical locations(Li et al. 2009; Xu et al. 2012). However, this has the potential to remove bonafide duplicates—those that came from different cDNA fragments, but due perhaps to the high expression level of a gene or deep sequencing for a library, came from the same location along a transcript. Moreover, we have found that libraries with high levels of PCR duplication tend to be less reliable when compared to replicates, and so prefer to simply discard those libraries, regenerating them from the initial RNA when possible.

However, there is not to our knowledge an accepted method to determine whether a sample has a high level of duplication. While examining GBrowse tracks for "blockiness" can qualitatively identify problems (as in Figure 1), it is both time consuming and not particularly rigorous. Noting the fraction of reads that map to unique locations is imperfect, as it will be sensitive to the depth of sequencing, and therefore potentially difficult to compare across libraries.

Comparing the number of unique read start sites to the total number of bases in a gene offers one potential way to quantify the amount of PCR duplication actually present in the library. We simulated drawing unique, independent positions from a 1.5kb transcript (the average *Drosophila* transcript size, according to Daines et al. (2011))., and noting the fraction of unique start sites. We recognize that this simulation is highly idealized: there is a position-dependent bias, most often favoring the $5\prime$ end of the read (Mortazavi et al. 2008; Hansen, Brenner, and Dudoit 2010; Picelli et al. 2014); not all read start sites are equally likely, as fragmentation followed size selection can lose the reads closest to either end; and in a stranded protocol (which we have not tested here) the "forward" read must, necessarily, come before the "reverse" read. Nevertheless, this captures the essence of the problem, and we will show that it closely matches real data.

For each gene, we calculated f, the fraction of bases in that gene that had a read start at that position. We plotted this quantity in figure 2 against the number of reads mapping to that gene divided by the gene length (that is,

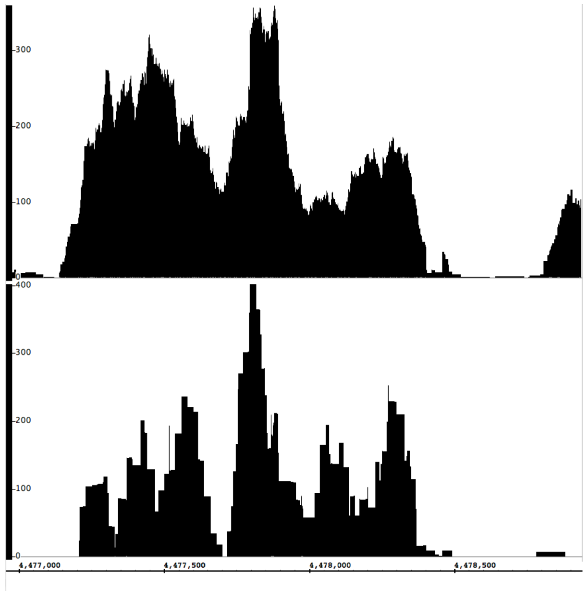

Figure 1: **GBrowse views of the same 2kb region of Chromosome 3R in two libraries with different levels of duplication.** Despite the similar number of reads in each sample, the lower library has much less information, due to a relatively high level of PCR duplication.

the total coverage, c). As expected, as the average coverage for each gene increased, the fraction of unique start sites increased as well, until the coverage approached 1x, at which point the available start sites became saturated.

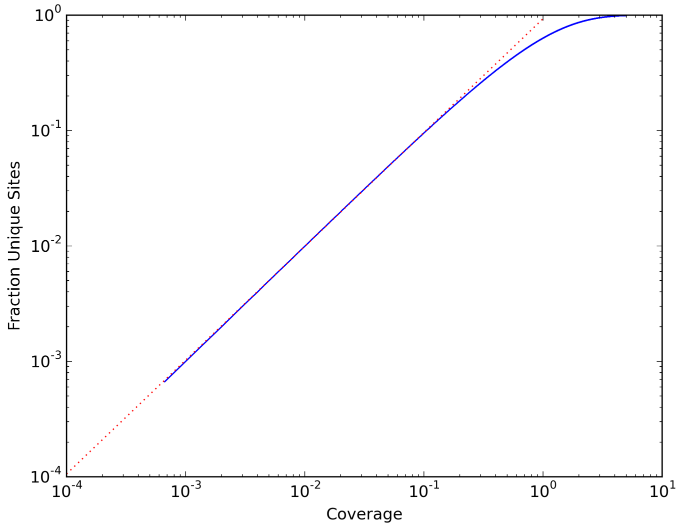

Figure 2: **Log-log plot of simulated unique sites as a function of coverage.** Simulated values are in blue, and the fit to the portion of the graph below 10% of the unique start sites is the dotted red line.

Crucially, when we plotted these on a log-log plot, the region below about 10% of the occupied start sites was approximately linear for at least 3 logs below that. The fit equation was:

$$\log_{10} f = m_{sim} \cdot \log_{10} c + b_{sim} \approx .986 \log_{10} c - 0.031 \tag{1}$$

or

$$f = 10^{b_{sim}} x^{m} \tag{2}$$

We expect the slope m to be slightly less than 1, to indicate that increasing the coverage should increase the fraction of start sites occupied, but with some chance of multiple, independent reads coming from the same location, even in the absence of duplication.

This leads to an easy interpretation of the score $B = 10^{b_{sim}-b}$ as the average level of PCR duplication. A lower intercept corresponds to a shift to

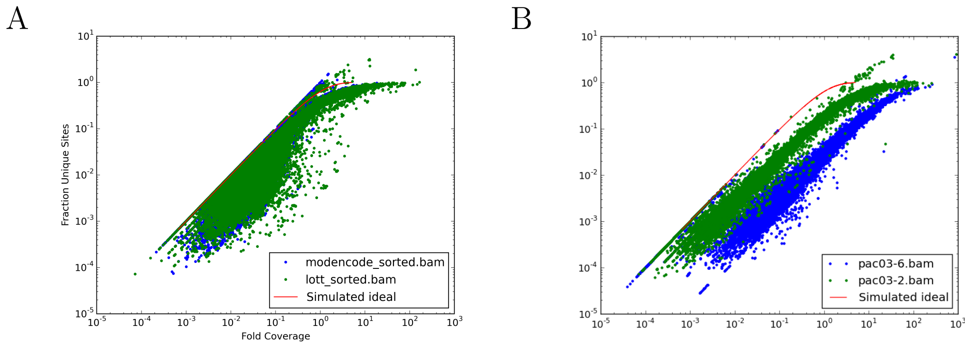

Figure 3: **Log-log plot of actual of fractions of unique sites vs coverage.** A) Previously published RNAseq data. Green points from modENCODE Consortium et al. 2010, with a B-score of 1.3. Blue points from Lott et al. 2011, with a B-score of 2.6. B) Unpublished, low-quality RNAseq data. Green points have a B-score of 5.7, blue points have a B-score of 36.5

the right on the plot, which in turn means that the coverage to yield a given number of unique sites must be higher.

When we calculated the B-score for samples from previously published datasets, both from our lab (Lott et al. 2011) and the modENCODE consortium (modENCODE Consortium et al. 2010), we found that the scores were all less than 3 (Figure 3A). By contrast, previous libraries from our lab that have been unpublished due to our lack of confidence that the data was not highly enriched for duplicates (including the lower panel of Figure 1) had B-scores in excess of 5 and up to 30 (Figure 3B). RNAseq data used in the main text of this study had B-scores as shown in table 1 in this document. Although in many cases they are higher than ideal, they are comparable to previously published data, and only one is greater than 4.3.

Protocol	% Virilis	b	m	B score
TruS	0	-0.6546	0.9195	4.2030
TruS	5	-0.4405	1.0584	2.5676
TruS	10	-0.5567	0.9998	3.3550
TruS	20	-0.5245	1.0224	3.1149
CT	0	-0.9669	0.8645	8.6285
CT	5	-0.5320	1.0386	3.1701
CT	10	-0.3784	1.1027	2.2254
CT	20	-0.5421	1.0335	3.2442
TotS	0	-0.2840	1.0871	1.7907
TotS	5	-0.2583	1.0769	1.6877
TotS	10	-0.4882	1.0199	2.8659
TotS	20	-0.4940	1.0202	2.9046
S2	0	-0.2139	1.1206	1.5238
S2	5	-0.1748	1.1303	1.3927
S2	10	-0.1586	1.1324	1.3416
S2	20	-0.1607	1.1302	1.3483
S2—2.5×	0	-0.5398	1.0036	3.2275
S2—2.5×	1	-0.6005	0.9771	3.7118
S2—2.5×	5	-0.5448	1.0038	3.2645
S2—2.5×	10	-0.6117	0.9713	3.8084
S2—2.5×	20	-0.5532	0.9987	3.3284
S2—5×	0	-0.5944	0.9815	3.6599
S2—5×	1	-0.6373	0.9620	4.0397
S2—5×	5	-0.5707	0.9842	3.4650
S2—5×	10	-0.5695	0.9951	3.4557
S2—5×	20	-0.5831	0.9930	3.5658

Table 1: Fit parameters and estimate duplication rate (B-score) of libraries used in the main text of this paper

We also simulated the fits for various sizes between 100bp and 10kb. Although the fit parameters did have a clear, increasing trend in response to increasing the simulated transcript size (Figure 4), the increase was small compared to the actual values. A variation of 0.005 in the intercept, which is used to calculate the B-score, corresponds to an actual difference of about 1%. We are thus not concerned about the choice of 1.5kb to simulate the ideal scenario.

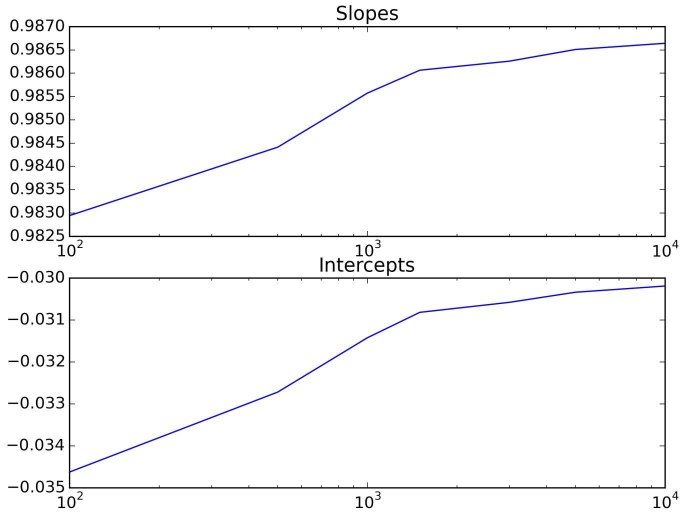

Figure 4: **Regression coefficients vs size of transcript.** Simulated at 100, 500, 1000, 1500, 3000, 5000, and 10,000 bp transcript sizes.

Simulation Code

```
from __future__ import division
from scipy import random
from numpy import (zeros, zeros_like, arange,
                   unique, mean, log10)
from scipy.stats import linregress
from progressbar import ProgressBar

avg_size = 1500.
```

```
n_reps = 100

regs = []
all_sizes = [100., 500., 1000., 1500., 3000., 5000., 10000.]

for avg_size in all_sizes:
    cs = arange(1, 5*avg_size)
    fs = zeros_like(cs)

    pb = ProgressBar()

    all_fracs = []
    for c in pb(cs):
        fracs = zeros(n_reps)
        for i in range(n_reps):
            fracs[i] = len(unique(
                random.randint(0, avg_size, c))
                         )/avg_size
            # Use randint to generate random read positions,
            # then count the number of unique start sites
            # and normalize by size of the transcript
            all_fracs.append((c/avg_size, fracs[i]))
        fs[c-1] = mean(fracs)

    print(’-’*30)
    print(avg_size)
    print(’-’*30)
    regs.append(linregress(log10(cs[fs<.1]/avg_size),
                           log10(fs[fs<.1])))
    print(regs[-1])
```

References

[1] Yuval Benjamini and Terence P Speed. “Summarizing and correcting the GC content bias in high-throughput sequencing.” In: *Nucleic Acids Research* 40.10 (May 2012), e72.

[2] Bryce Daines et al. “The Drosophila melanogaster transcriptome by paired-end RNA sequencing.” In: *Genome Research* 21.2 (Feb. 2011), pp. 315–324.

[3] Kasper D Hansen, Steven E Brenner, and Sandrine Dudoit. “Biases in Illumina transcriptome sequencing caused by random hexamer priming.” In: *Nucleic Acids Research* 38.12 (July 2010), e131.

[4] Heng Li et al. "The Sequence Alignment/Map format and SAMtools." In: *Bioinformatics (Oxford, England)* 25.16 (Aug. 2009), pp. 2078–2079.

[5] Susan E Lott et al. "Noncanonical compensation of zygotic X transcription in early Drosophila melanogaster development revealed through single-embryo RNA-seq." In: *PLoS Biology* 9.2 (2011), e1000590.

[6] Lira Mamanova et al. "FRT-seq: amplification-free, strand-specific transcriptome sequencing." In: *Nature Methods* 7.2 (Jan. 2010), pp. 130–132.

[7] modENCODE Consortium et al. "Identification of functional elements and regulatory circuits by Drosophila modENCODE." In: *Science (New York, N.Y.)* 330.6012 (Dec. 2010), pp. 1787–1797.

[8] Ali Mortazavi et al. "Mapping and quantifying mammalian transcriptomes by RNA-Seq." In: *Nature Methods* 5.7 (July 2008), pp. 621–628.

[9] Simone Picelli et al. "Full-length RNA-seq from single cells using Smart-seq2." In: *Nature Protocols* 9.1 (Jan. 2014), pp. 171–181.

[10] Haibin Xu et al. "FastUniq: a fast de novo duplicates removal tool for paired short reads." In: *PLoS ONE* 7.12 (2012), e52249.
