# Supplementary figures and images for: Low-cost, low-input RNA-seq protocols perform nearly as well as high-input protocols
Source: PeerJ. 2015 Mar 26;3:e869. doi: 10.7717/peerj.869 (PMC4380159; doi:10.7717/peerj.869)

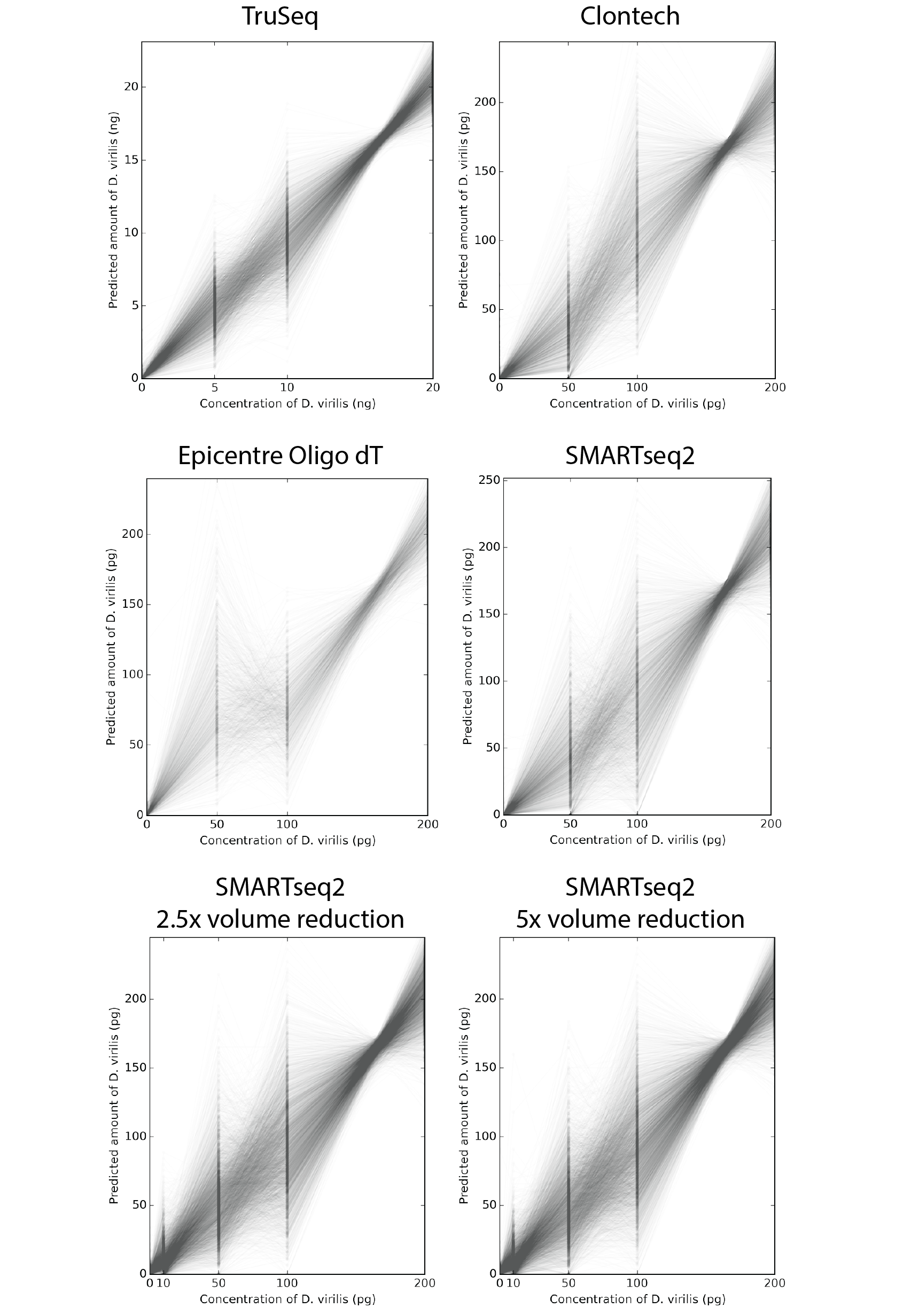

Supplement: Supplemental Information 3 [file peerj-03-869-s004.png]

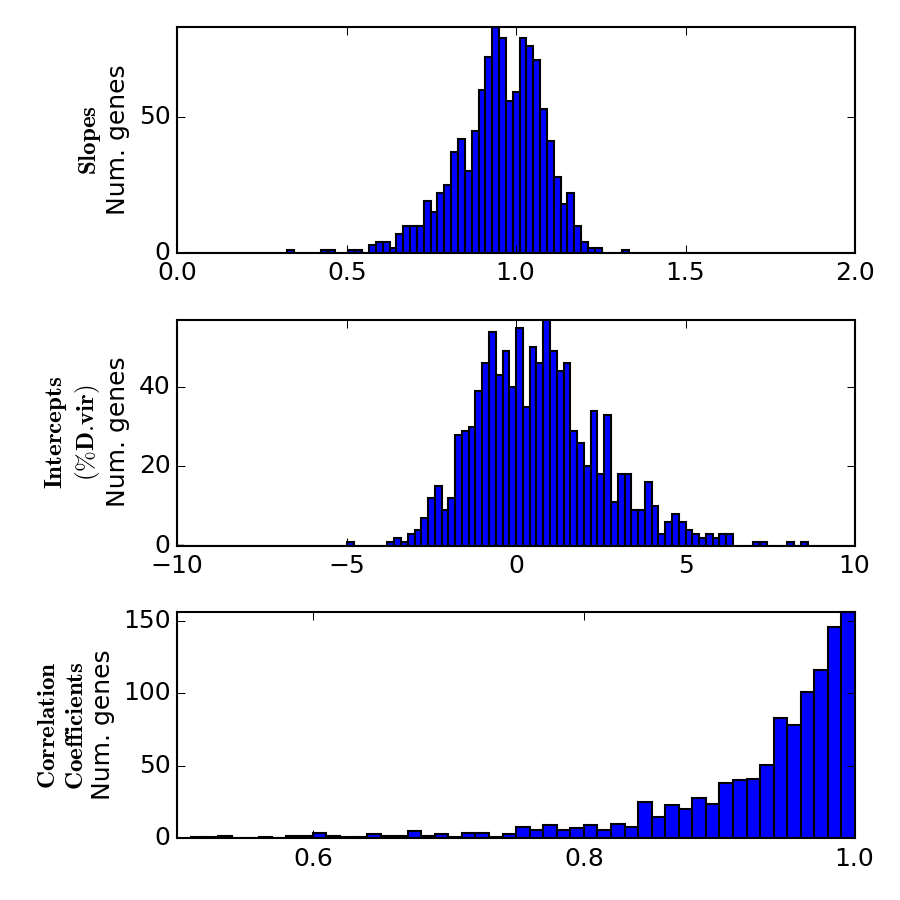

Supplement: Supplemental Information 4 — The distributions are practically indistinguishable from the corresponding distribution for the TruSeq data, in 1B. [file peerj-03-869-s005.png]
